# Broadband All-Polymer Phototransistors with Nanostructured Bulk Heterojunction Layers of NIR-Sensing n-Type and Visible Light-Sensing p-Type Polymers

**DOI:** 10.1038/srep16457

**Published:** 2015-11-13

**Authors:** Hyemi Han, Sungho Nam, Jooyeok Seo, Chulyeon Lee, Hwajeong Kim, Donal D. C. Bradley, Chang-Sik Ha, Youngkyoo Kim

**Affiliations:** 1 Organic Nanoelectronics Laboratory, School of Applied Chemical Engineering, Kyungpook National University, Daegu 702-701, Republic of Korea; 2 Center for Plastic Electronics and Department of Physics, Blackett Laboratory, Imperial College London, London SW7 2AZ, United Kingdom; 3 Research Institute of Advanced Energy Technology, Kyungpook National University, Daegu 702-701, Republic of Korea; 4 Department of Polymer Science and Engineering, Pusan National University, Busan 609-735, Republic of Korea; 5 Department of Engineering Science, Division of Mathematical, Physical and Life Sciences, University of Oxford, 9 Parks Road, Oxford OX1 3PD, United Kingdom; 6 Department of Physics, Division of Mathematical, Physical and Life Sciences, University of Oxford, 9 Parks Road, Oxford OX1 3PD, United Kingdom

## Abstract

We report ‘*broadband light-sensing*’ all-polymer phototransistors with the nanostructured bulk heterojunction (BHJ) layers of visible (VIS) light-sensing electron-donating (p-type) polymer and near infrared (NIR) light-sensing electron-accepting (n-type) polymer. Poly[{2,5-bis-(2-ethylhexyl)-3,6-bis-(thien-2-yl)-pyrrolo[3,4-c]pyrrole-1,4-diyl}-co-{2,2′-(2,1,3-benzothiadiazole)]-5,5′-diyl}] (PEHTPPD-BT), which is synthesized via Suzuki coupling and employed as the n-type polymer, shows strong optical absorption in the NIR region (up to 1100 nm) in the presence of weak absorption in the VIS range (400 ~ 600 nm). To strengthen the VIS absorption, poly(3-hexylthiophene) (P3HT) is introduced as the p-type polymer. All-polymer phototransistors with the BHJ (P3HT:PEHTPPD-BT) layers, featuring a peculiar nano-domain morphology, exhibit typical p-type transistor characteristics and efficiently detect broadband (VIS ~ NIR) lights. The maximum corrected responsivity (without contribution of dark current) reaches up to 85 ~ 88% (VIS) and 26 ~ 40% (NIR) of theoretical responsivity. The charge separation process between P3HT and PEHTPPD-BT components in the highest occupied molecular orbital is proposed as a major working mechanism for the effective NIR sensing.

Photodetectors have greatly contributed to the development of science and technology and to the paradigm change of human cultures, since they have been widely applied from a simple sensor for various instruments to an image sensor for biomedical diagnosis systems and personal electronic devices (smart phones etc.)^1^,^2^,^3^,^4^. To date, most of commercial photodetectors have been fabricated with inorganic semiconductors because of well-established infrastructures in manufacturing of inorganic devices even on a nanoscale^5^,^6^. In particular, inorganic photodetectors are able to easily detect near infrared (NIR) light, due to a sophisticated band gap engineering of inorganic (semiconducting) materials^7^,^8^, which delivered tremendous applications for optical communications, biomedical imaging, etc^9^,^10^,^11^. However, inorganic photodetectors have a fundamental drawback in terms of flexibility/lightweight and low temperature process owing to the intrinsic nature of inorganic materials even though the process temperature is being reduced in part by applying wet processes^12^,^13^,^14^.

In this context, organic photodetectors have been introduced because organic photo-sensing layers (typically 20 ~ 100 nm) can be easily fabricated at low (room) temperatures by employing a variety of wet-coating processes including spin-coating, slot-die coating, gravure coating, ink-jet printing, etc^15^,^16^,^17^,^18^,^19^. Organic photodetectors can be classified into two types: organic photodiodes (OPDIs) and organic phototransistors (OPTRs). OPDIs have a simple diode structure in which organic photo-sensing layers are sandwiched between two electrodes, while OPTRs have a triode structure in which additional gate (G) electrode (compared to the diode type) plays a critical role in amplifying and/or addressing photocurrent signals. In particular, organic photo-sensing layers can be coated in a large-area of flexible plastic substrates, which delivers OPTRs with a big advantage for low-cost fabrication of large-area two-dimensional array type detectors (image sensors) in the coming flexible electronics era.

To date, various studies have been reported for OPTRs that are fabricated with a single type of organic photo-sensing layers [i.e., individual p-type (electron-donating) or n-type (electron-accepting) organic semiconductors]^20^,^21^,^22^,^23^,^24^,^25^. However, relatively less attention has been paid to OPTRs with bulk heterojunction (BHJ) photo-sensing layers of p-type and n-type organic components^26^,^27^,^28^,^29^, of which benefits have been well demonstrated in organic solar cells^30^,^31^,^32^. Moreover, no study has been reported on the OPTRs with all-polymer BHJ layers which can detect broadband lights from full visible (VIS) to NIR regions.

In this work, we fabricated *broadband* OPTRs with all-polymer BHJ layers that consist of VIS light-sensing p-type polymer and NIR light-sensing n-type polymer. Poly(3-hexylthiophene) (P3HT) was used as the visible light-sensing p-type component in consideration of its strong absorption in the VIS range up to 650 nm. As the NIR light-sensing n-type polymer, poly[{2,5-bis-(2-ethylhexyl)-3,6-bis-(thien-2-yl)-pyrrolo[3,4-c]pyrrole-1,4-diyl}-co-{2,2′-(2,1,3-benzothiadiazole)]-5,5′-diyl}] (PEHTPPD-BT), which was synthesized via the Suzuki coupling reaction, was employed because of its strong absorption of NIR photons up to 1100 nm. Flexible *broadband* all-polymer phototransistors were fabricated with the present P3HT:PEHTPPD-BT BHJ layers and poly(ethylene naphthalate) (PEN) substrates, which demonstrated low-voltage operations below −5 V. In order to understand how the present OPTRs could detect such a broadband light from VIS to NIR ranges, all-polymer BHJ (P3HT:PEHTPPD-BT) layers were investigated with high resolution transmission electron microscopy (HRTEM), atomic force microscopy (AFM) and synchrotron radiation grazing incidence angle X-ray diffraction (GIXD) measurements.

## Synthesis, Energy Band and Nanostructure of PEHTPPD-BT

**Figure 1:**
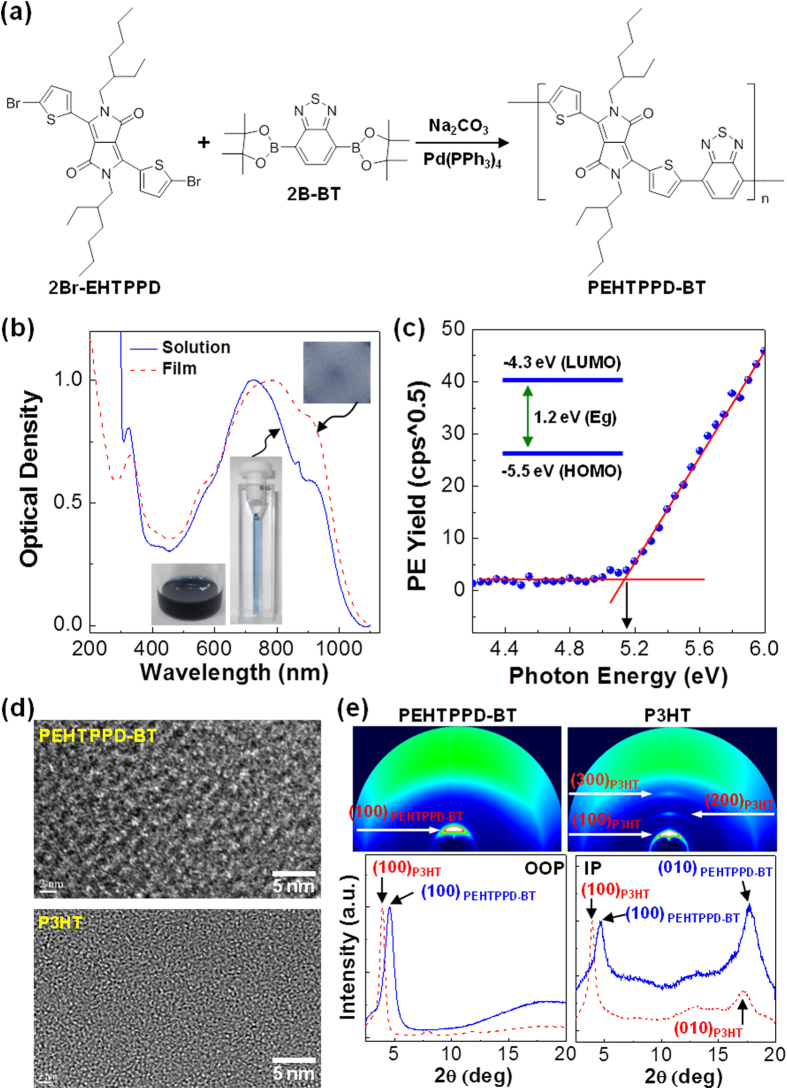
Scheme, optical properties and nanostructures. (**a**) Synthesis of PEHTPPD-BT from 2Br-EHTPPD and 2B-BT via Suzuki coupling reaction. (**b**) Optical absorption spectra of PEHTPPD-BT in solution (o-dichlorobenzene) and thin film (thickness = ca. 200 nm) (see inset photographs). (**c**) Photoelectron (PE) yield spectrum of the PEHTPPD-BT film coated on glass substrate (inset: flat energy band diagram for PEHTPPD-BT). (**d**) HRTEM images for the pristine PEHTPPD-BT layer (top) and the pristine P3HT layer (bottom). (**e**) 2D GIXD images (top) and 1D GIXD profiles (bottom) for the two pristine polymers.

The PEHTPPD-BT polymer was synthesized via the Suzuki coupling reaction, as depicted in Fig. 1a, from 3,6-bis(5-bromothiophen-2-yl)-2,5-bis(2-ethylhexyl) pyrrolo[3,4-c]pyrrole-1,4(2H,5H)-dione (2Br-EHTPPD) and 2,1,3-benzothiadiazole-4,7-bis(boronic acid pinacol ester) (2B-BT) in the presence of tetrakis(triphenylphosphine)palladium(0) (Pd(PPh_3_)_4_) and sodium carbonate (Na_2_CO_3_) as a catalyst in the mixture solvent of tetrahydrofuran (THF) and deionized water (H_2_O). The detailed synthesis procedures are described in methods section. As shown in Fig. 1b, the optical absorption spectrum of the PEHTPPD-BT polymer covered a wide VIS-NIR range (up to 1100 nm). In particular, the PEHTPPD-BT film showed a noticeably increased optical density in the NIR region (>800 nm) in the presence of bathochromic shift (ca. 100 nm) at the maximum peak, which indicates the PEHTPPD-BT chains are supposed to effectively stack each other in the solid state. Interestingly, the optical absorption spectrum of the PEHTPPD-BT film was almost unchanged by thermal annealing at 150 °C for 30 min (see Figure S1), reflecting high glass transition temperature >150 °C. The highest occupied molecular orbital (HOMO) energy of the PEHTPPD-BT film was calculated ~5.5 eV after calibration from the photoelectron (PE) yield spectrum (see Fig. 1c)^33^,^34^. Then the lowest unoccupied molecular orbital (LUMO) energy of the PEHTPPD-BT film (~4.3 eV) was calculated from both HOMO energy and optical band gap energy (Eg = 1.2 eV) (taken from the onset point of optical absorption spectrum in Fig. 1b). The completed energy band diagram is given in the inset of Fig. 1c.

Interestingly, as shown in the HRTEM image (Fig. 1d), the nanostructure of the PEHTPPD-BT film, which corresponds to a random polycrystalline structure and is clearly different from that of the P3HT film, disclosed that the crystal size of the PEHTPPD-BT chains is larger than that of the P3HT chains. The relatively large polycrystalline nanostructure of PEHTPPD-BT can be attributed to the distorted structure of the PEHTPPD-BT chains owing to the steric hindrance between EHTPPD units and BT units (see Figure S2). The crystalline structure of the PEHTPPD-BT films was also confirmed from the 2D GIXD image in Fig. 1e, which shows the intense Debye ring for the (100) diffraction of PEHTPPD-BT. The 1D GIXD profiles deliver that the (100) diffraction peak in the out-of-plane (OOP) direction is located at 4.6° for PEHTPPD-BT and 3.9° for P3HT. These diffraction angles provide *d*-spacing values of *d* = 1.42 nm for PEHTPPD-BT and *d* = 1.66 nm for P3HT. This information implies that the PEHTPPD-BT chains are much closely stacked each other in the *a-a* direction of molecular structure (see Figure S3), which can be ascribed to the distorted and bended chain structure of PEHTPPD-BT as measured previously for the P3HT chains in the presence of other components^35^,^36^. Similarly, the wider (100) diffraction angle (4.6°) for PEHTPPD-BT than P3HT was measured in the 1D GIXD profiles in the in-plane (IP) direction. In particular, the (010) diffraction of PEHTPPD-BT (*d* = 0.37 nm) was more intense than that of P3HT (*d* = 0.38 nm), indicative of more pronounced side-on (edge-on) orientation of PEHTPPD-BT chains (in the presence of local distortion of repeating units) than P3HT chains. The ideal stacking structure of PEHTPPD-PD chains is illustrated in Figure S3.

## Transistor Performances in the Dark Condition

**Figure 2:**
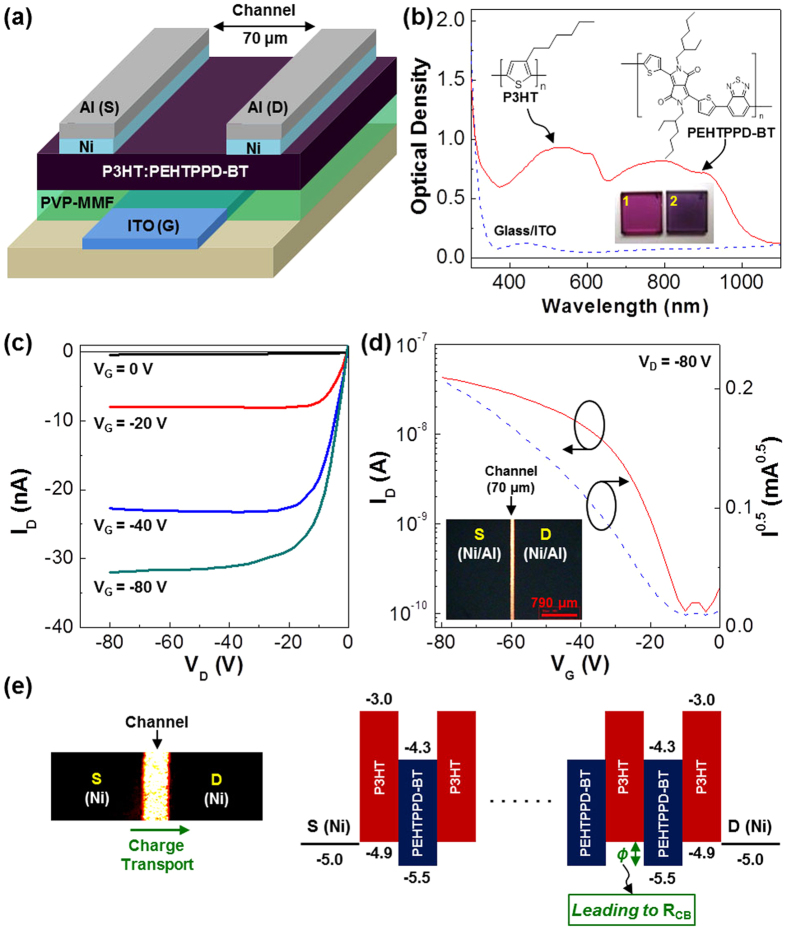
OPTR structure, broadband absorption and transistor performances. (**a**) Device structure of OPTRs with all-polymer BHJ (P3HT:PEHTPPD-BT) layer. (**b**) Optical absorption spectra of the ITO-glass substrate and the P3HT:PEHTPPD-BT layer-coated ITO-glass substrate (see the photograph ‘2’ compared with the photograph ‘1’ of the P3HT layer-coated ITO-glass substrate). (**c**) Output characteristics of OPTR with the P3HT:PEHTPPD-BT layer in the dark. (**d**) Transfer characteristics of OPTR with the P3HT:PEHTPPD-BT layer in the dark (V_D_ = −80 V): The inset optical microscope (OM) image shows the channel area for the OPTR fabricated in this work, where S and D denote the source electrode and the drain electrode, respectively. (**e**) Illustration for the charge blocking resistance (R_CB_) owing to the HOMO energy barrier (*ϕ*) (see the enlarged OM image on the left as a guide for charge transport direction).

As shown in Fig. 2a, the present OPTRs were fabricated using the P3HT:PEHTPPD-BT (1:1 by weight) BHJ layers (see details in methods section) by employing a basic transistor structure of bottom gate (G) and top source/drain (S/D) electrodes. In order to secure the efficient charge (hole) injection from the S/D electrodes into the BHJ layer, the 10 nm-thick Ni layer was inserted between the BHJ layer and the Al electrode. As shown in Fig. 2b, the P3HT:PEHTPPD-BT layer exhibited a broadband absorption covering from 300 nm to 1100 nm (see the film color in the inset photograph). A particular attention is paid to the fact that the P3HT component did effectively complement the low optical density region of the PEHTPPD-BT component at a wavelength of ca. 400 ~ 600 nm, while the absorption for the NIR region was strengthened by the PEHTPPD-BT component in the present BHJ layer (see Figure S4 for the individual absorption spectrum).

As shown in Fig. 2c, the present devices exhibited typical output curves with a clear saturation current when drain voltage (V_D_) increased negatively. In addition, the output curves were systematically shifted toward a higher (negative) drain current (I_D_) direction as gate voltage (V_G_) increased negatively, indicating a p-type transistor for the present devices. We note that the drain current was also significantly increased (negatively) as V_G_ increased negatively at a fixed drain voltage (V_D_ = −80 V), as seen from the transfer curve in Fig. 2d. The threshold voltage (V_TH_) was measured as ca. −13 V, while the hole mobility (μ_h_) was calculated as ~3 × 10^−4^ cm^2^/Vs by saturation regime equation as described in Figure S5. Interestingly, the dark performance of the present OPTRs with the BHJ (P3HT:PEHTPPD-BT) layers is quite comparable to that of typical organic field-effect transistors (OFETs) with the P3HT channel layers which were fabricated in the same way as for the OPTRs (see Figure S6)^37^,^38^,^39^. This result supports that the charge transport in the P3HT:PEHTPPD-BT layer is not significantly limited by the co-existence of the two polymers. In more detail, the P3HT:PEHTPPD-BT layer is supposed to retain a phase-segregated nanomorphology (sufficient for efficient charge transport) without a huge charge-blocking resistance (R_CB_), which could be caused by the large HOMO energy barrier (*ϕ*) in the case of an ideal (molecular level) mixing state between PEHTPPD-BT and P3HT chains (see Fig. 2e). The detailed nanomorphology will be discussed later^40^.

## Phototransistor Performances under Illumination with VIS & NIR Lights

**Figure 3:**
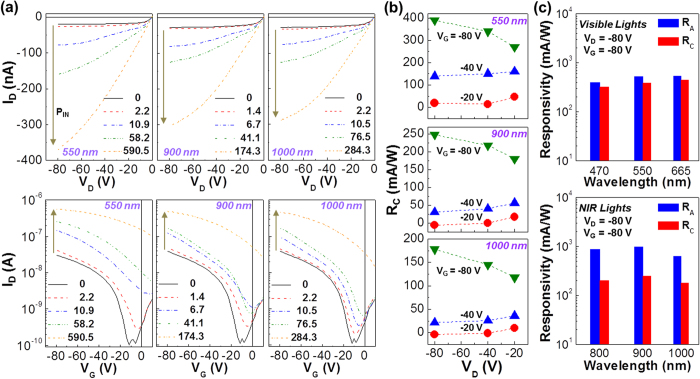
Phototransistor performances under VIS and NIR lights. (**a**) Output (top; V_G_ = −80 V) and transfer (bottom; V_D_ = −80 V) characteristics (selected) for OPTRs with the P3HT:PEHTPPD-BT layers according to the incident light density (power density, P_IN_) under illumination with VIS (550 nm) and NIR (900 and 1000 nm) lights. (**b**) Corrected responsivity (R_C_) as a function of drain voltage (V_D_) at three different gate voltages under illumination with VIS and NIR lights. (**c**) Comparison between apparent responsivity (R_A_) and R_C_ under illumination with VIS (top) and NIR (bottom) lights. Note that R_A_ and R_C_ values were taken from the transfer curves in (**a**) and Figures S7–S8 at similar range of P_IN_ (1.4 ~ 2.2 μW/cm^2^).

The output curves of the OPTRs at a fixed gate voltage (V_G_ = −80 V) were measured under illumination with VIS (470 nm, 550 nm and 665 nm) and NIR (800 nm, 900 nm and 1000 nm) lights. As shown in Fig. 3a (top: selected wavelengths) and Figure S7 (all wavelengths), the drain current in the output curves was certainly increased as the incident light intensity (P_IN_) increased irrespective of wavelength. In addition, the drain current under illumination was remarkably increased (negatively) as V_D_ increased (negatively), suggesting strong amplification of sensing signals by adjusting drain voltage at a fixed gate voltage. Considering the photocurrent generation under illumination with NIR lights that cannot excite the P3HT component at all, the PEHTPPD-BT component in the BHJ layer is supposed to transport holes in the present OPTRs. As observed from the transfer curves at a fixed drain voltage (V_D_ = −80 V) (Fig. 3a bottom), the drain current was drastically increased as the incident light intensity increased for both VIS and NIR lights. The similar trend was measured for the transfer curves at lower drain voltages (V_D_ = −20 V and −40 V) (see Figure S8).

From the transfer curves in Fig. 3a, the responsivity of devices was calculated according to various combinations of drain and gate voltages. The apparent responsivity (R_A_), which is the ratio of the drain current measured under illumination to the incident light density after unit area calibration (see details in methods section), was quite sensitive to both gate and drain voltages irrespective of wavelengths (see Figure S9a). To clarify the exact responsivity without the influence of dark current density (J_DD_), the corrected responsivity (R_C_ = (J_DP_ − J_DD_)/P_IN_) was calculated by subtracting J_DD_ from the drain current under illumination (J_DP_). As summarized in Fig. 3b for the representative wavelengths (see Figure S9b for full wavelengths), the R_C_ trend was basically similar to the R_A_ trend in terms of the voltage dependence but the R_C_ value was relatively lower than the R_A_ value. A particular attention is paid to the huge dependence of the R_C_ value on the gate voltage rather than the drain voltage, indicative of the effective signal amplification by the gate voltage control. As shown in Fig. 3c, the maximum R_C_ value was 320 ~ 450 mA/W (VIS) and 170 ~ 250 mA/W (NIR) at V_G_ = −80 V and V_D_ = −80 V, which correspond to 85 ~ 88% and 26 ~ 40% of a theoretical responsivity, respectively (see Figures S10 and S11 for the calculation of responsivity depending on the device geometry*—we note that calculation of responsivity should be very careful because abnormal values overwhelming theoretical limits for typical photodetectors could be obtained if the direction (cross-sectional area) of drain current and incident light is not properly calibrated - this is why extremely high responsivity values have been reported in the couple of previous reports*). Such higher R_C_ values under illumination with VIS lights can be attributed to two reasons: (1) The VIS lights could be absorbed by both P3HT and PEHTPPD-BT components whereas the NIR lights were absorbed only by the PEHTPPD-BT component (see Fig. 1b); (2) The n-type PEHTPPD-BT component in the BHJ structure might help efficient charge separation from the excitons generated in the P3HT component so that the resulting photo-generated holes could contribute to the improved charge transport in addition to the holes generated by the electric field effect. The detailed mechanism will be discussed later.

## Flexible Plastic Phototransistors for Sensing Broadband Lights

**Figure 4:**
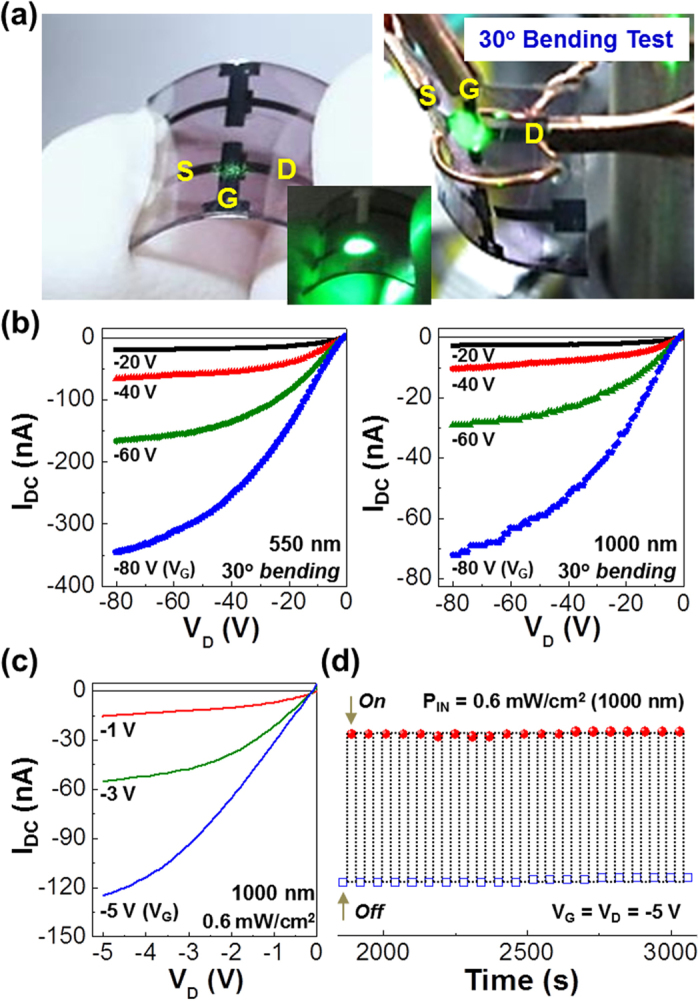
Flexible plastic OPTR performances. (**a**) Photographs for flexible plastic OPTRs with the P3HT:PEHTPPD-BT layers, which are bended at an angle of ~30° for the measurement of phototransistor performances (note that the green light was roughly illuminated as a guide for the channel part). (**b**) Output characteristics (upon bending at 30°) for the flexible OPTRs with the P3HT:PEHTPPD-BT layers under illumination with VIS (550 nm) and NIR (1000 nm) lights: I_DC_ stands for the drain current created purely by light without dark current. (**c**,**d**) Low-voltage operation (upon bending at 30°) of the optimized flexible plastic OPTRs under illumination with NIR (1000 nm) lights: (**c**) output curves and (**d**) repeated on/off modulation.

Based on the results from the OPTRs with ITO-glass substrates, flexible plastic OPTRs with the P3HT:PEHTPPD-BT layers were fabricated by employing poly(ethylene naphthalate) (PEN) substrates (see the detailed fabrication process in methods section). As shown in Fig. 4a, the present plastic OPTRs were flexible and bent well with fingers. In order to examine the performance of plastic OPTRs, the output curves were measured upon bending at an angle of 30° (see Fig. 4a right). As shown in Fig. 4b, the pure drain current (I_DC_ = I_DP_ − I_DD_) by light illumination, which is given by subtracting the dark drain current (I_DD_) from the light drain current under illumination (I_DP_), was clearly measured for both VIS (550 nm) and NIR (1000 nm) lights. In particular, the pure drain current was noticeably increased by increasing either V_G_ or V_D_, which indicates excellent amplification of signals in the present plastic OPTRs. Further optimization process enabled the present plastic OPTRs to be operated at low voltages (−1 V ~ −5 V) for the detection of NIR light (see Fig. 4c). In addition, the optimized plastic OPTRs were considerably stable in sensing the NIR light, as supported from the repeated measurement by on/off modulation of NIR light (see Fig. 4d).

## Nanostructures & Nanomorphology in the P3HT:PEHTPPD-BT Layers

**Figure 5:**
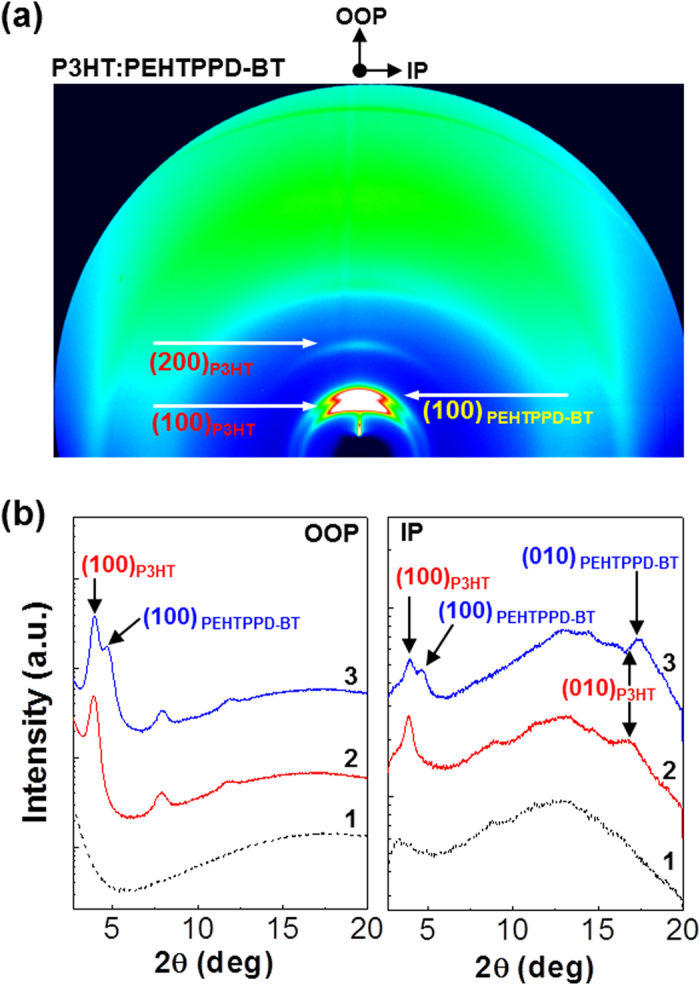
BHJ nanostructures. (**a**) 2D GIXD image for the P3HT:PEHTPPD-BT layer coated on the PVP-MMF/ITO-glass substrate. (**b**) 1D GIXD profiles in the OOP (left) and IP (right) directions for the PVP-MMF layer coated on the ITO-glass (1), the pristine P3HT layer coated on the PVP-MMF/ITO-glass (2), and the P3HT:PEHTPPD-BT layer coated on the PVP-MMF/ITO-glass (3).

To understand how the present OPTRs could sense both VIS and NIR lights, the nanostructure of the BHJ (P3HT:PEHTPPD-BT) layers was first examined with synchrotron radiation GIXD measurements. As shown in the 2D GIXD image (Fig. 5a), two separated Debye rings in the (100) diffraction were measured in the presence of single (200) diffraction ring (for P3HT). One of the two individual (100) diffractions can be assigned to the P3HT component at the smaller angle, while another can be designated to the PEHTPPD-BT component at the larger angle (see Fig. 1e for the diffraction images for the pristine polymer films). The 1D GIXD profiles in the OOP direction (Fig. 5b) confirm that the two separated (100) diffraction peaks at 3.93° and 4.67° correspond to the P3HT component and the PEHTPPD-BT component, respectively. This result delivers valuable information that the two polymer components were not well mixed in a molecular level but made an individual crystalline domain leading to a phase-segregation. Here we need to note that the P3HT component in the BHJ layer showed higher order diffraction peaks ((200) and (300)) in the OOP direction, which supports that the P3HT chains underwent a pronounced ***a-a*** geometry stacking^41^ even in the presence of the PEHTPPD-BT component in the present BHJ layer. Considering the (010) diffraction peak position in Fig. 5b (right), the PEHTPPD-BT chains might make a predominant ***b-b*** stacking in the presence of some P3HT ***b-b*** stacking^42^. This result reflects that both P3HT and PEHTPPD-BT chains made an edge-on stacking in the present BHJ layer, which is of crucial importance to secure in-plane charge transport^43^,^44^.

**Figure 6:**
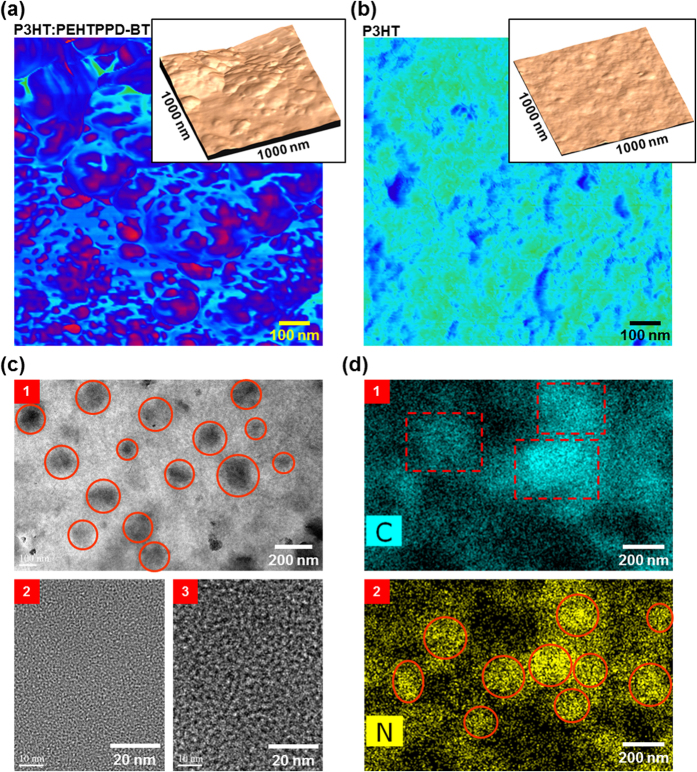
BHJ nanomorphology. (**a**,**b**) Phase-mode AFM images (1 μm × 1 μm) for the P3HT:PEHTPPD-BT layer (**a**) and the pristine P3HT layer (**b**), which were coated on the PVP-MMF/ITO-glass substrates (insets: height-mode images). (**c**) TEM images for the P3HT:PEHTPPD-BT layer (film) (1: low magnification image; 2: bright part image; 3: dark part image): Note that the ‘2’ and ‘3’ images were measured separated by focusing on each part after increasing the magnification of HRTEM system. (**d**) STEM images for chemical mapping of carbon (1) and nitrogen (2) atoms in the P3HT:PEHTPPD-BT layer (film): Note that the atom distribution is quite broad because of the transmission mode in the STEM measurement which delivers the whole atom information in the thickness direction.

In order to clarify the nanostructures obtained by the GIXD measurements, the nanomorphology of the BHJ layer was investigated through the AFM and HRTEM measurements. As shown in the AFM images (Fig. 6), the P3HT:PEHTPPD-BT layer exhibited a distinct nanomorphology with random nano-domains on the surface (Fig. 6a), which is quite different from the pristine P3HT layer with much smoother surface (Fig. 6b). This surface morphology supports that the two polymer components were not ideally mixed in a molecular level but phase-segregated on a nanoscale in the BHJ layer. The size of nano-domains can be measured from <10 nm to 50 nm in the presence of bigger domains that could be formed by aggregation of small nano-domains. The TEM image (Fig. 6c) confirms that such nano-domains were randomly distributed in the BHJ layer and featured darker contrast in the electron diffraction. Further examination disclosed that the nano-domain parts did actually correspond to the PEHTPPD-BT component, while the surrounding region was composed of the P3HT component (see ‘2’ and ‘3’ in Fig. 6c). However, as observed from the scanning TEM (STEM) images in Fig. 6d, the PEHTPPD-BT component was not solely located in the nano-domains but was distributed broadly (even small amount) over the whole BHJ layer when it comes to the nitrogen mapping profile with dispersive distributions (see “2” in Fig. 6d).

## Proposed Nanostructure and Working Mechanism

**Figure 7:**
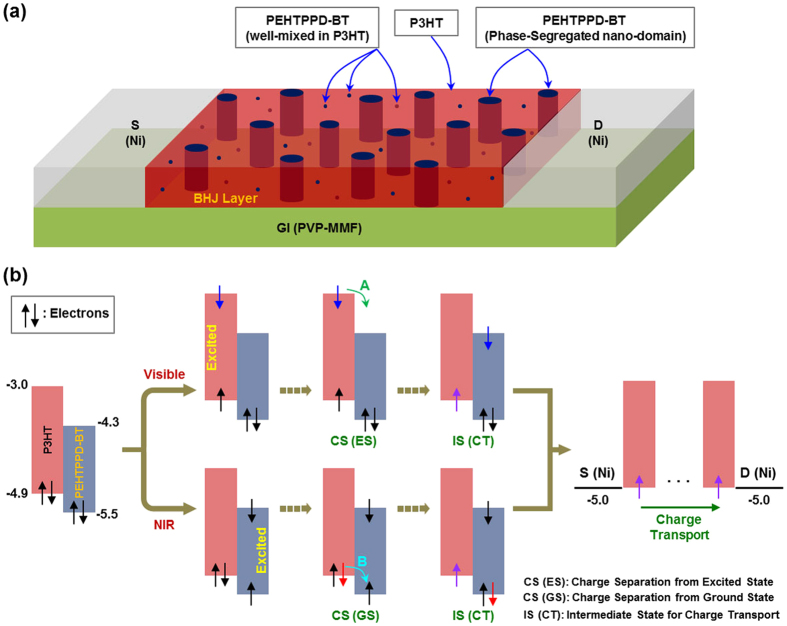
Proposed BHJ nanostructure and working mechanism. (**a**) Illustration for the nanostructure proposed for the present P3HT:PEHTPPD-BT layer in the (simplified) OPTR geometry, which features the PEHTPPD-BT nano-domains in the presence of the PEHTPPD-BT chains and small aggregates dispersed in the P3HT matrix. (**b**) Proposed working mechanism for the present OPTRs with the P3HT:PEHTPPD-BT layers in order to successfully detect both VIS and NIR lights: ‘CS (ES)’, ‘CS (GS)’ and IS (CT)’ denote ‘charge separation from an excited state’ (see ‘A’), ‘charge separation from a ground state’ (see ‘B’), and ‘intermediate state for charge transport’, respectively. Note that the flat energy band structure for the BHJ layer only is displayed here and the unit ‘eV’ is omitted in order to avoid crowding the diagram.

Based on the GIXD/AFM/TEM results, a possible (idealized) nanostructure for the BHJ (P3HT:PEHTPPD-BT) layer is proposed as illustrated in Fig. 7a (note that the size and shape of nano-domains might be different in the real BHJ layer). As marked in the illustration, the major part of the PEHTPPD-BT component is considered to exist in the nano-domains in the presence of its minor part that could be dispersed in a shape of single polymer chains and/or aggregates smaller than the nano-domains in the P3HT matrix. Considering the proposed nanostructure for the BHJ layer, working mechanism can be divided into two ways, depending on the wavelength ranges (VIS and NIR) as described in Fig. 7b. VIS lights can be assumed to mainly excite the P3HT component to make excitons (see top parts in Fig. 7b) (note that the PEHTPPD-BT component can also absorb VIS light but its absorption is relatively weaker than that of the P3HT component as discussed in Fig. 1b). These excitons lead to individual charges after charge separation process (see ‘A’ in ‘CS (ES)’), which secures an intermediate state for charge transport (see ‘IS (CT)’). This intermediate state means that photo-generated holes (unpaired single electrons) are formed in the HOMO level of the P3HT component by optical excitation, which serves as an active pathway for charge transport in addition to another intermediate state formed by electric field (namely, ‘field-effect’). In contrast, NIR lights can selectively excite the PEHTPPD-BT component (see bottom parts in Fig. 7b) because the P3HT component cannot absorb the NIR lights. Next, the electron pairs in the HOMO level of the P3HT component are subjected to charge separation process (see ‘B’ in ‘CS (GS)’) by the influence of photo-generated holes (unpaired single electrons) in the PEHTPPD-BT component (note that the driving force for charge separation is attributed to the HOMO band offset between P3HT and PEHTPPD-BT (ΔE ~ 0.6 eV)), which results in the intermediate state for charge transport (see ‘IS (CT)’). Thus holes in the P3HT component are ready for efficient charge transport upon illumination with NIR lights. Here it is worthy to note that holes in the HOMO level of the PEHTPPD-BT component are expected to partly involve in the (direct) charge transport, even though their contribution is considered to be extremely limited when it comes to the isolated PEHTPPD-BT nano-domains in the BHJ layer as well as the relatively large energy barrier for charge injection to the HOMO level of the PEHTPPD-BT component from the Ni electrode.

In summary, organic phototransistors (OPTRs) with all-polymer BHJ layers of P3HT and PEHTPPD-BT were fabricated by employing a transistor configuration of bottom gate and top source/drain electrodes. The PEHTPPD-BT polymer synthesized in this work exhibited a coarse polycrystalline nanostructure and a wide optical absorption range from UV-VIS to NIR. A broadband optical absorption profile was measured for all-polymer BHJ layers (P3HT:PEHTPPD-BT = 1:1 by weight). All-polymer phototransistors fabricated delivered typical p-type transistor characteristics in the dark, while huge photocurrent signal was measured upon illumination with VIS and NIR lights. The maximum R_C_ reached ~450 mA/W (VIS) and ~250 mA/W (NIR), which correspond to 85 ~ 88% (VIS) and 26 ~ 40% (NIR) of theoretical responsivities. Flexible plastic OPTRs with the P3HT:PEHTPPD-BT layers were operated at low voltages (−1 V ~ −5 V) and could detect VIS and NIR lights even at a bended state. In-depth measurements disclosed that the present BHJ layers possess a particular nanostructure with randomly-distributed PEHTPPD-BT nano-domains in the P3HT matrix. The (excited state) charge separation process, which occurs from the HOMO level of the P3HT component to that of the PEHTPPD-BT component in the present OPTRs, is proposed as a major working mechanism for the effective sensing of NIR lights leading to achieving ‘*broadband light-sensing*’ all-polymer OPTR technology.

## Methods

### Materials and Solutions

Poly(3-hexylthiophene) (P3HT) (regioregularity = 93 ~ 95%; weight-average molecular weight = 50 ~ 70 kDa; polydispersity index = 1.4–1.6) was used as received from Solaris Chemistry Inc. (Canada). 3,6-bis(5-bromothiophen-2-yl)-2,5-bis(2-ethylhexyl) pyrrolo [3,4-c] pyrrole-1,4(2 H,5 H)-dione (2Br-EHTPPD) was purchased from Solarmer Energy (China). 2,1,3-Benzothiadiazole-4,7-bis(boronic acid pinacol ester) (2B-BT), sodium carbonate (Na_2_CO_3_), tetrakis(triphenylphosphine)palladium(0) (Pd(PPh_3_)_4_) were purchased from Sigma-Aldrich (USA). Poly(4-vinylphenol) (PVP), methylated poly(melamine-co-formaldehyde) (MMF), nickel (Ni) and aluminum (Al) were purchased from Sigma-Aldrich (USA). Tetrahydrofuran (THF), chlorobenzene (CB), 1,2-dichlorobenzene (o-DCB), propylene glycol monomethyl ether acetate (PGMEA) were purchased from TCI (Japan) and Sigma-Aldrich (USA). Solutions of pristine P3HT and PEHTPPD-BT polymers were prepared using o-DCB at a solid concentration of ca. 20 mg/ml, while binary blend solutions (P3HT:PEHTPPD-BT = 1:1 by weight) of the two polymers were prepared using the same solvent at a solid concentration of 20 ~ 30 mg/ml. Binary solutions of PVP and MMF were prepared using PGMEA solvent at a solid concentration of 225 mg/mL (PVP:MMF = 1:1.25 by weight). These solutions were subject to vigorous stirring at 60 °C for 3 days.

### Synthesis of PEHTPPD-BT

The PEHTPPD-BT polymer was synthesized via a palladium-catalyzed Suzuki coupling reaction. 2Br-EHTPPD (0.3 mmol), 2B-BT (0.3 mmol), Na_2_CO_3_ (2 M), and Pd(PPh_3_)_4_ (5 mol%) were added to a flat bottom three-necked flask in a glove box (nitrogen environment). After taking out the flask containing reactants and catalysts, the flask was flushed with argon gas. Next, THF (7.5 ml), deionized water (DIW, 6 ml) and trioctylmethylammonium chloride (Aliquat^®^ 336) were added to the mixture, followed by degassing with argon and heating at 85 °C for 48 h. Then Pd(PPh_3_)_4_ (2.5 mol%) was injected sequentially into the reaction mixture for additional activation. The full reaction was carried out for 24 h. To terminate reaction, the flask was cooled down to room temperature and diluted with CB. Solvents were removed using a rotary evaporator leading to the concentrated solutions, which were then dissolved thoroughly in chlorobenzene, followed by extraction of catalysts using DIW. The organic phase in the separation process was filtrated with a filter paper (pore size = 1 μm). The filtrates were concentrated and precipitated into methanol (150 ml). The resulting solid was washed with various organic solvents (methanol, ethanol, isopropyl alcohol, hexane and toluene) repeatedly to remove low molecular weight fractions and other impurities. The precipitates were filtered finally and dried in vacuum, leading to the PEHTPPD-BT polymer (a deep-blue solid, 70 mg, yield: 35%). The molecular weight of PEHTPPD-BT was ca. 31.6 kDa, which was measured with a matrix-assisted laser desorption ionization-time of flight-mass spectrometer (MALDI-TOF-MS, Bruker, USA). The solubility test result according to the kind of solvent is given in Figure S12 (note that o-DCB exhibited good solubility compared to other solvents). We note that a similar polymer with different alkyl groups has been published^45^.

### Thin Film and Device Fabrication

Bottom-gate-type OPTRs were fabricated using glass substrates with a stripe (1 mm × 12 mm) of indium-tin oxide (ITO, ~20 Ω/cm^2^) gate electrode, which was patterned with typical photolithography/etching process. The PVP-MMF precursor films were spin-coated on top of the cleaned ITO-glass substrates, followed by prebaking at 120 °C for 10 min and thermal cross-linking at 250 °C for 30 min, leading to thermoset-type PVP-MMF layers (thickness (t) = 600 nm). Next, the BHJ layers (P3HT:PEHTPPD-BT, t = 100 nm) were spin-coated on the PVP-MMF layer at 1500 rpm for 60 s and soft-baked at 60 °C for 15 min. These samples were loaded into a vacuum chamber installed inside an argon-filled glove box system, followed by thermal annealing at 150 °C for 30 min. Finally, 10 nm-thick Ni and 60 nm-thick Al metallic layers were sequentially deposited through a shadow mask to form source/drain electrodes, defining a transistor geometry (channel length = 70 μm; channel width = 3 mm). For the fabrication of top-gate-type plastic OPTRs, 70 nm-thick Al and 10 nm-thick Ni layers were deposited on the PEN substrates (t = 200 μm) through a shadow mask to form source/drain electrodes (channel length = 70 μm; channel width = 2 mm). The BHJ layers (P3HT:PEHTPPD-BT, t = 20 nm) were spin-coated on the PEN substrates with Ni/Al electrodes at 2000 rpm for 60 s, followed by soft-baking at 80 ^°^C for 15 min. Next, poly(methyl methacrylate) (PMMA) layers (t = 850 nm) were spin-coated on the BHJ layers at 1200 rpm for 60 s and soft-baked at 80 °C for 15 min. These samples were loaded into a vacuum chamber, followed by deposition of 70 nm-thick Al gate electrodes. The fabricated devices were stored inside the argon-filled glove box before measurements in order to avoid attacks by moisture and oxygen. To confirm the reproducibility (NIR detection) of device results, two different synthesis batches were applied for the fabrication of devices (see Figure S13).

### Measurements and Analysis

Optical absorption spectra of solutions and films were measured using a UV-VIS spectrometer (Optizen 2120+, Mecasys), while a photoelectron spectrometer (AC2, Hitachi High Tech) was used for the measurement of ionization potentials. The nanostructures of pristine and BHJ layers were examined using a synchrotron radiation grazing incidence angle X-ray diffraction system (9A beamline, Pohang Accelerator Laboratory, Republic of Korea). The wavelength and incidence angle of X-ray was 0.11352 nm and 0.12°, respectively. The surface morphology of pristine and BHJ layers was investigated using an atomic force microscope (Nanoscope IIIa, Digital Instruments), while the crystal nanostructure of film samples were measured using a high resolution transmission electron microscope (HRTEM, Titan G2 ChemiSTEM Cs Probe, FEI Company). The atom composition profile of film samples was measured using a scanning transmission electron microscope (STEM, Titan G2 ChemiSTEM Cs Probe, FEI Company). The transistor performances were measured using a semiconductor parameter analyzer (2636B and 4200SCS, Keithley). The hole mobility of OFETs was calculated in the saturation regime from the slope of transfer curves using the equation, 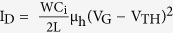, where 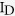 is drain current, 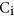 is capacitance per unit area of gate dielectric layer, and 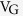
and 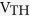 are gate voltage and threshold voltage, respectively (see also Figure S5). To measure phototransistor characteristics, the channel area of OPTRs was illuminated with a monochromatic light passed through a monochromator (CM110, Spectral Products) from a white light generated by a Tungsten-Halogen lamp (150 W, ASBN-W, Spectral Products). The incident light intensity (P_IN_) was measured with a calibrated Si photodiode (818-UV, Newport, USA). The exact calculation of responsivity is given in Figure S10.

## Additional Information

**How to cite this article**: Han, H. *et al.* Broadband All-Polymer Phototransistors with Nanostructured Bulk Heterojunction Layers of NIR-Sensing n-Type and Visible Light-Sensing p-Type Polymers. *Sci. Rep.* **5**, 16457; doi: 10.1038/srep16457 (2015).

## Supplementary Material

Supplementary Information

Supplementary Video
