# Supplementary material for: Broadband All-Polymer Phototransistors with Nanostructured Bulk Heterojunction Layers of NIR-Sensing n-Type and Visible Light-Sensing p-Type Polymers
Source: Sci Rep. 2015 Nov 13;5:16457. doi: 10.1038/srep16457 (PMC4643233; doi:10.1038/srep16457)
Supplement: Supplementary Information [file srep16457-s1.pdf]

[Supporting Information]

Broadband All-Polymer Phototransistors with Nanostructured Bulk Heterojunction Layers of NIR-Sensing n-Type and Visible Light-Sensing p-Type Polymers

Hyemi Han[1], Sungho Nam[1,2], Jooyeok Seo[1], Chulyeon Lee[1], Hwajeong Kim[1,3*], Donal D. C. Bradley[2], Chang-Sik Ha[4], and Youngkyoo Kim[1*]

[1]Organic Nanoelectronics Laboratory, School of Applied Chemical Engineering, Kyungpook National University, Daegu 702-701, Republic of Korea

[2]Center for Plastic Electronics and Department of Physics, Blackett Laboratory, Imperial College London, London SW7 2AZ, United Kingdom

[3]Research Institute of Advanced Energy Technology, Kyungpook National University, Daegu 702-701, Republic of Korea

[4]Department of Polymer Science and Engineering, Pusan National University, Busan 609-735, Republic of Korea

*Email) ykimm@knu.ac.kr, khj217@knu.ac.kr

This supporting information contains following data:

Figure S1. Comparison of optical absorption spectra

Figure S2. Energy minimized molecular structures (3D)

Figure S3. Illustration for possible stacking structures of the PEHTPPD-BT polymer chains

Figure S4. Comparison of optical absorption spectra between the PEHTPPD-BT layer and the P3HT layer

Figure S5. Demonstration for the calculation of hole mobility from the transfer curve of the OPTR

Figure S6. Output (top) and transfer (bottom) curves for the OFET with the pristine P3HT layer

Figure S7. Output (a) and transfer (b) curves for the OPTRs with the P3HT:PEHTPPD-BT BHJ layers

Figure S8. Transfer curves for the OPTRs with the P3HT:PEHTPPD-BT BHJ layers

Figure S9. Change of responsivity for the OPTRs with the P3HT:PEHTPPD-BT BHJ layers

Figure S10. Discrepancy of theoretical responsivity in OPTR

Figure S11. Replotted responsivity from Fig. S9 in the case of uncalibrated unit areas

Figure S12. Solubility of PEHTPPD-BT polymer

Figure S13. Reproducibility of device results for NIR detection

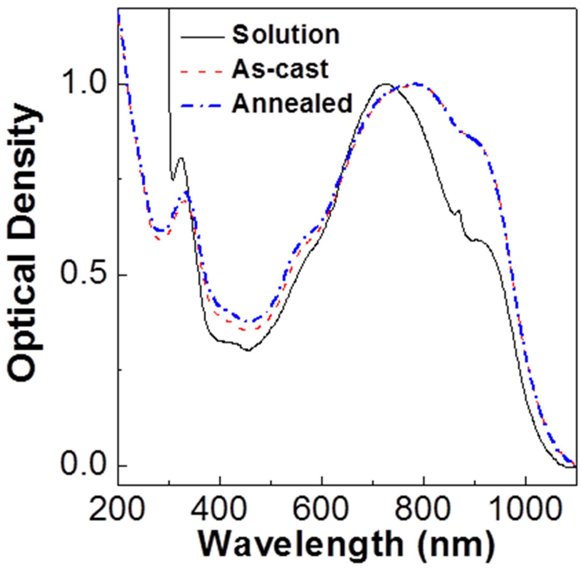

Figure S1. Comparison of optical absorption spectra: (black solid line) PEHTPPD-BT solution (o-dichlorobenzene), (red dashed line) PEHTPPD-BT layer coated on quartz substrate (soft-baking at 60 °C for 15 min), and (blue dash-dot line) annealed PEHTPPD-BT layer after coating on quartz substrate (thermal annealing at 150 °C for 30 min).

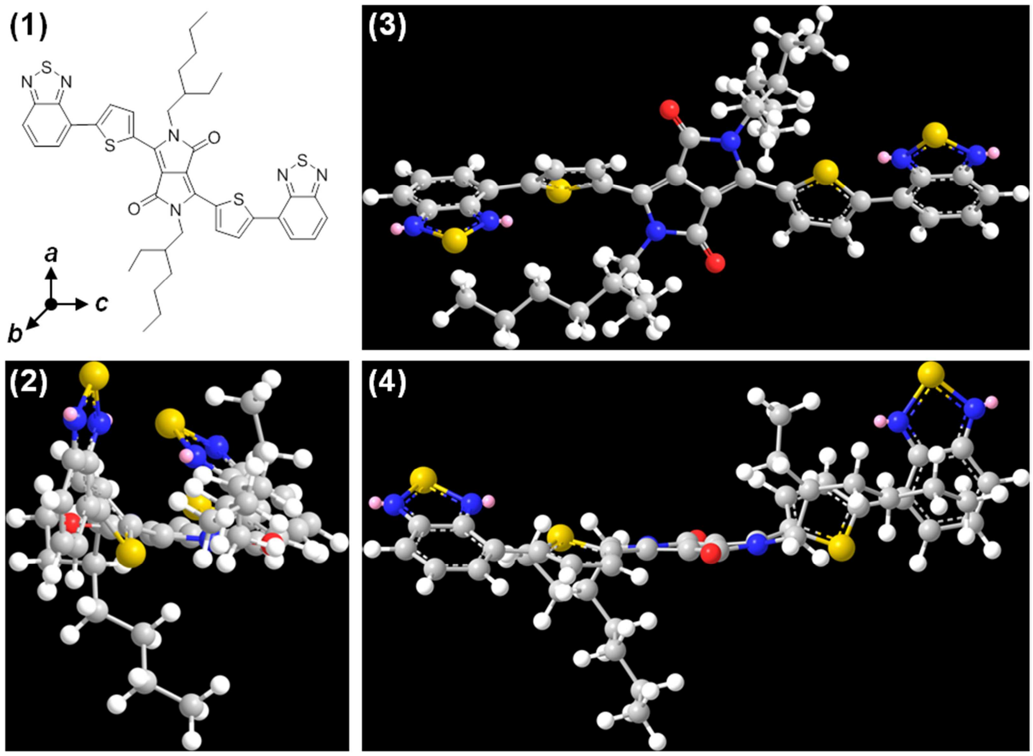

Figure S2. Energy minimized molecular structures (3D) for the moiety with one EHTPPD unit and two BT units according to the viewing directions: (1) 2D structure, (2~4) 3D structures. Note that the BT units are distorted from the plane of the EHTPPD unit (see attached video file: S2-Video.avi).

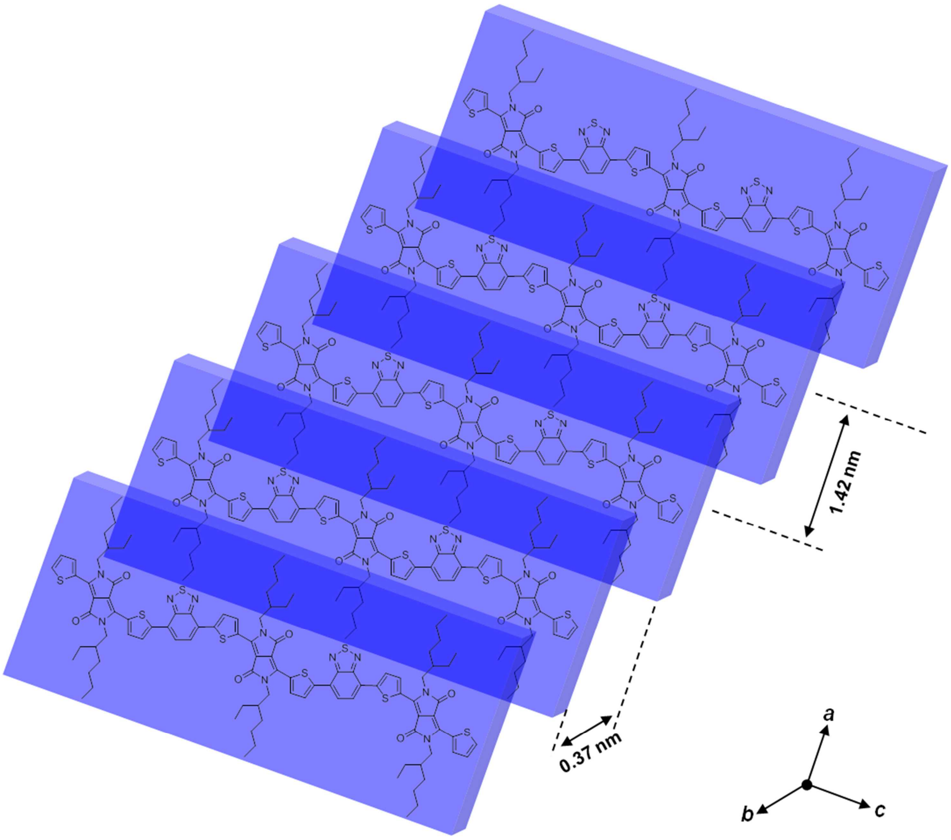

Figure S3. Illustration for possible stacking structures of the PEHTPPD-BT polymer chains: Note that the angle among each axis (a, b, c) is assumed as 90° though it seems to be distorted in the present drawing owing to the inevitably twisted display of the chemical structure (ideal). The stacking distances for ***a-a*** and ***b-b*** directions are taken from the GIXD results in Figure 1.

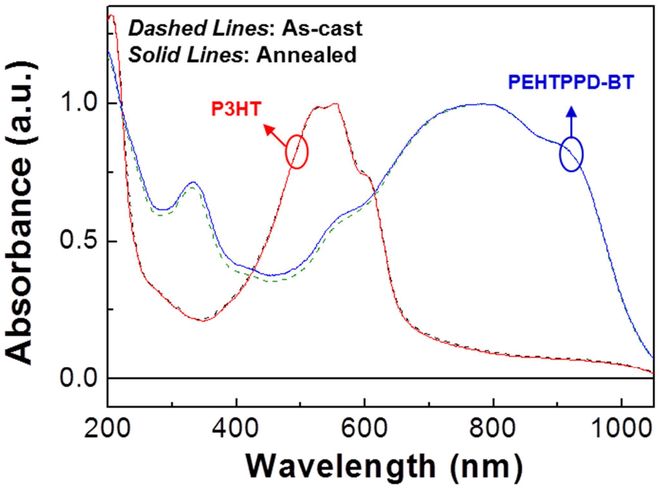

Figure S4. Comparison of optical absorption spectra between the PEHTPPD-BT layer and the P3HT layer, which were separately coated on quartz substrates. Note that the absorption spectra were almost unchanged even after thermal annealing at 150 ^oC for 30 min.

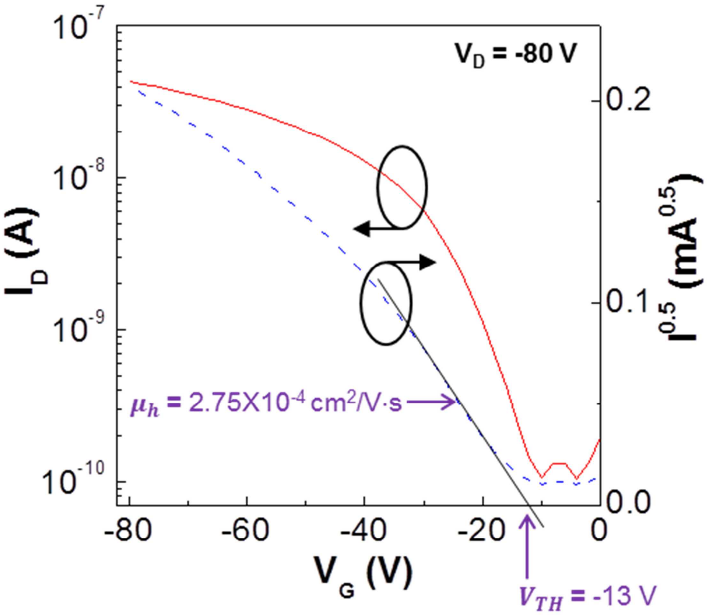

$$I_D = \frac{WC_i}{2L} \mu_h (V_G - V_{TH})^2$$

I_D : Drain current
L : Channel length
W : Channel width
C_i : Capacitance per unit area of the gate dielectric layer
μ_h : Hole mobility
V_G : Gate voltage
V_{TH} : Threshold voltage

Figure S5. Demonstration for the calculation of hole mobility from the transfer curve of the OPTR with the present P3HT:PEHTPPD-BT BHJ layer. The resulting hole mobility was ~3×10^{-4} cm^2/Vs at V_D = -80 V.

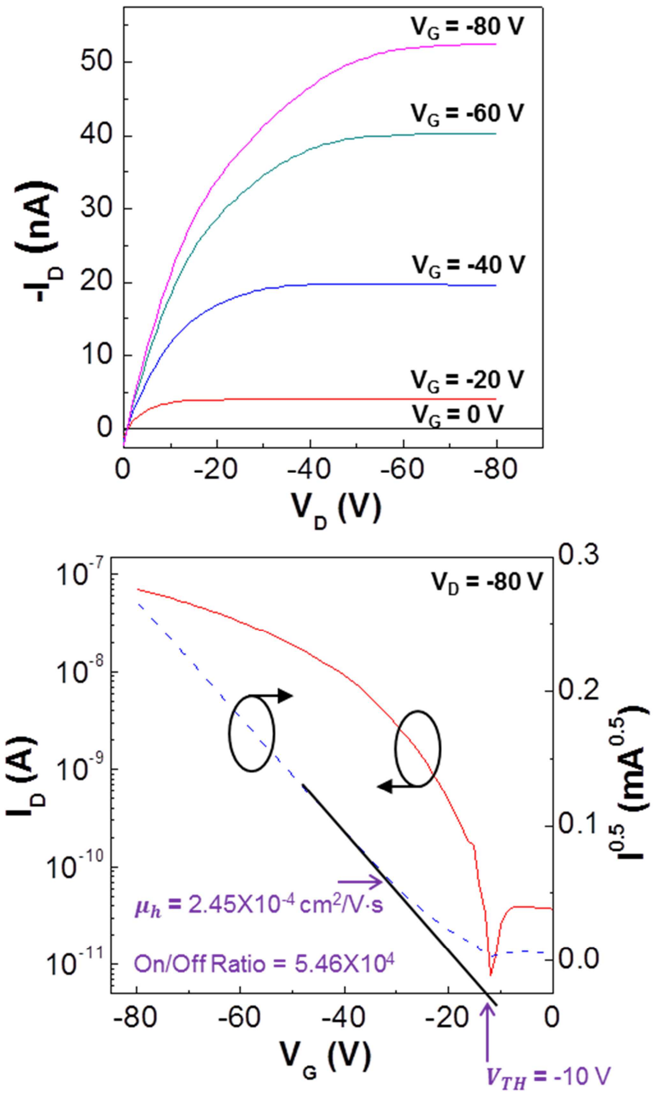

Figure S6. Output (top) and transfer (bottom) curves for the OFET with the pristine P3HT layer which was fabricated in the same way (condition) as for the OPTRs with the P3HT:PEHTPPD-BT BHJ layers. The resulting hole mobility was 2.45×10^{-4} cm^2/Vs at V_D = -80 V. The thickness of PVP-MMF, P3HT, Ni and Al was 600 nm, 50 nm, 10 nm and 60 nm, respectively.

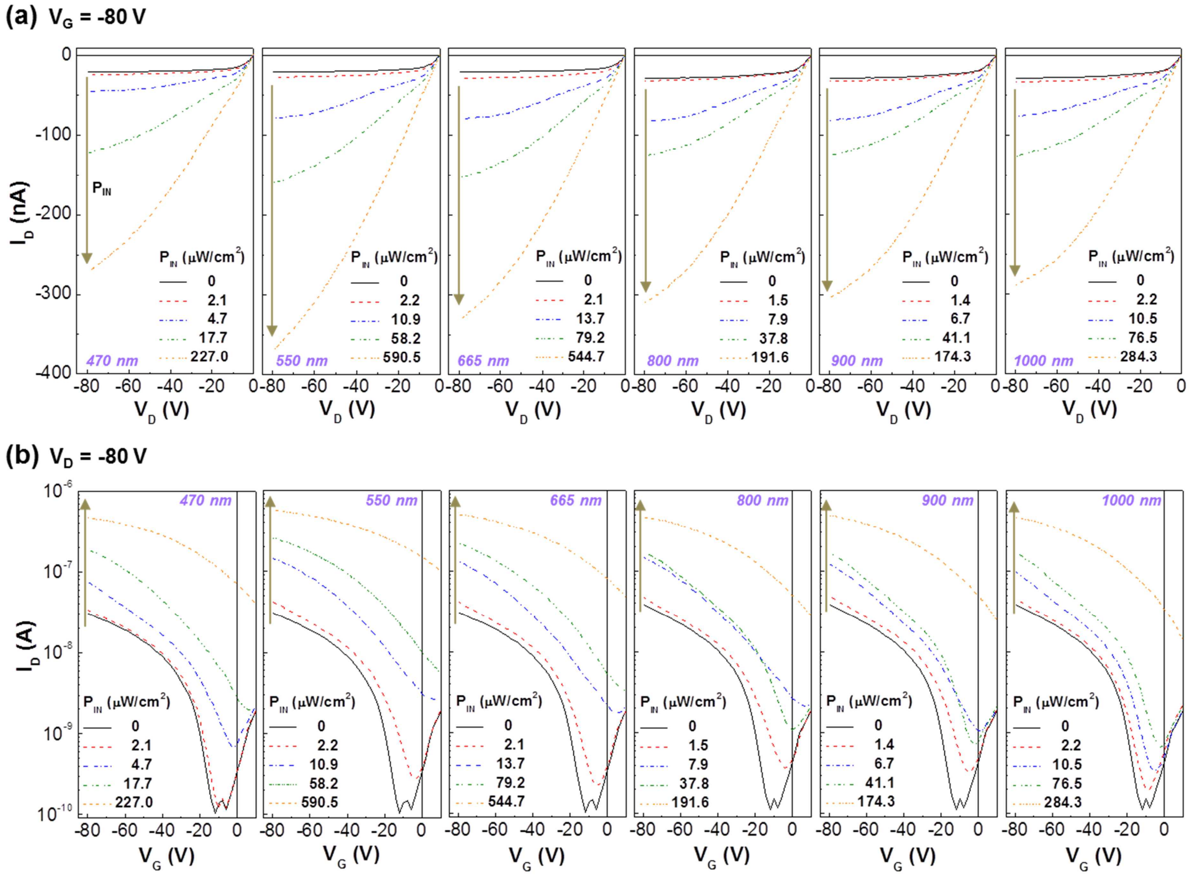

Figure S7. Output (a) and transfer (b) curves for the OPTRs with the P3HT:PEHTPPD-BT BHJ layers under illumination with VIS (470, 550, 665 nm) and NIR (800, 900, 1000 nm) lights according to the incident light intensity (P_{IN}).

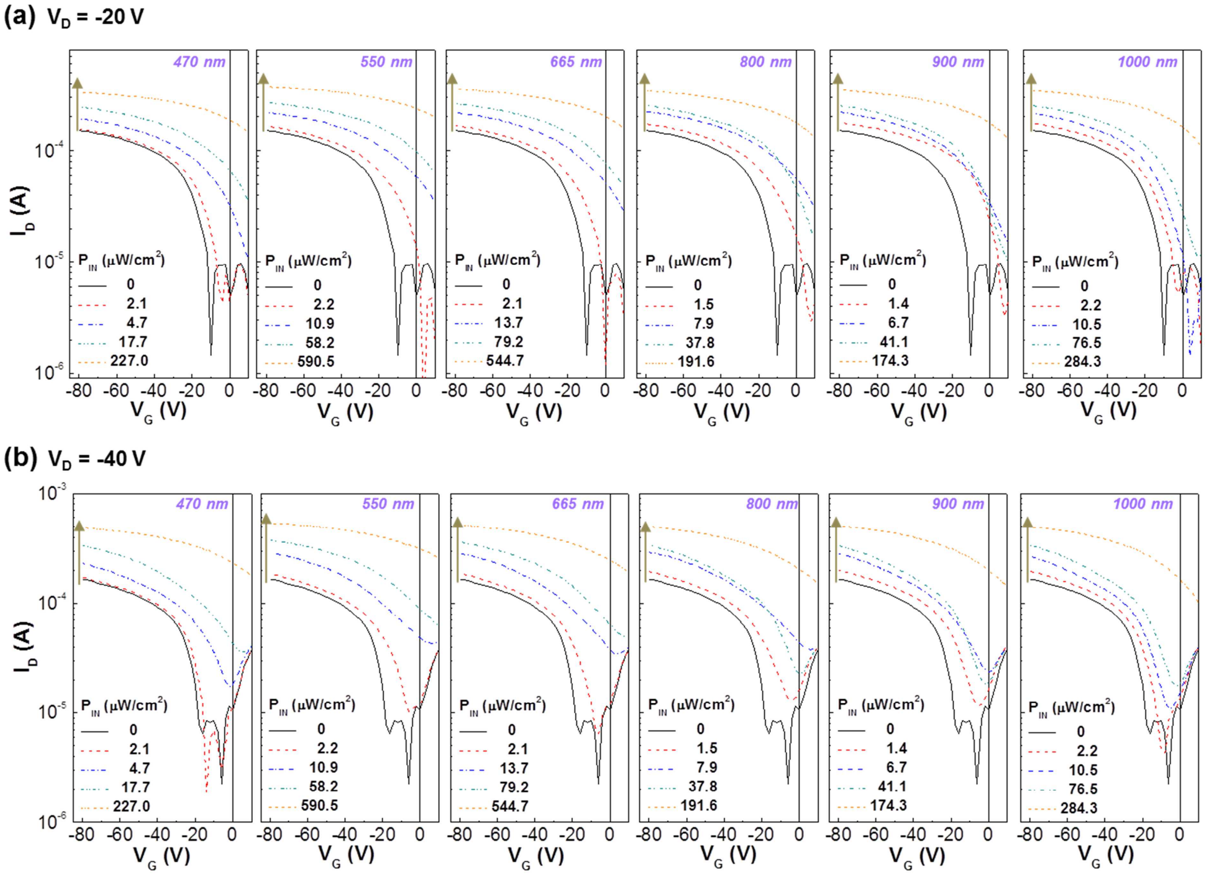

Figure S8. Transfer curves (selected at lower drain voltages) for the OPTRs with the P3HT:PEHTPPD-BT BHJ layers under illumination with VIS (470, 550, 665 nm) and NIR (800, 900, 1000 nm) lights according to the incident light intensity (P_{IN}): (a) V_D = -20 V, (b) V_D = -40 V.

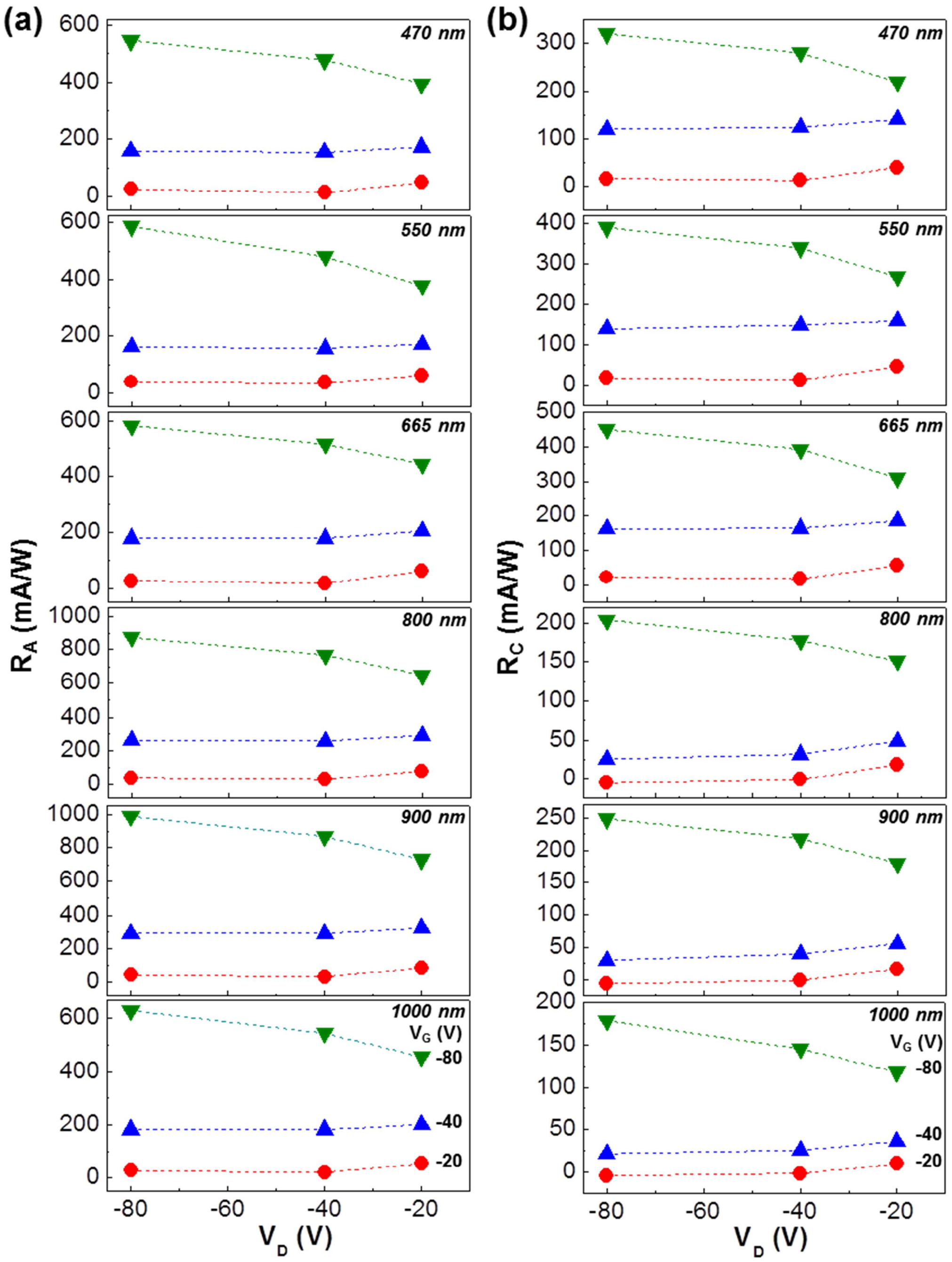

Figure S9. Change of responsivity (a: R_A and b: R_C) for the OPTRs with the P3HT:PEHTPPD-BT BHJ layers under illumination with VIS (470, 550, 665 nm) and NIR (800, 900, 1000 nm) lights according to the drain voltages at a fixed gate voltage (V_G = -20 V, -40 V and -80 V).

(a)

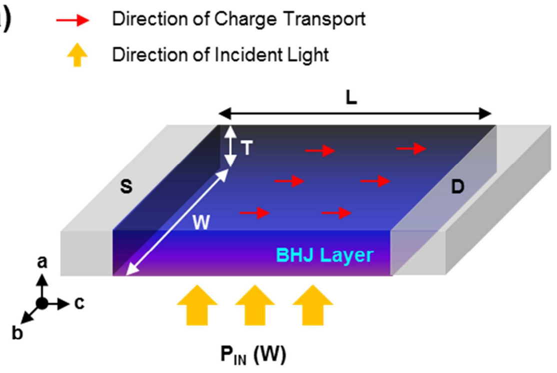

$$J = \frac{I}{Area\_1} = \frac{I}{T \times W} \left[\frac{\mathrm{A}}{\mathrm{cm}^2}\right]$$

$$P_{IN} = \frac{P}{Area\_2} = \frac{P}{L \times W} \left[\frac{\mathrm{W}}{\mathrm{cm}^2}\right]$$

$$R_C = \frac{J}{P_{IN}} = \frac{I}{P} \times \frac{L}{T} \left[\frac{\mathrm{A}}{\mathrm{W}}\right]$$

I : Current (without dark current)
J : Current density (I/Area_1)
P : Incident light intensity
P_{IN} : Incident light density (P/Area_2)
T : Thickness of the active layer
W : Channel width
L : Channel length
R_C : Corrected responsivity

(b)

Wavelength (nm)	Theoretical R_C (A/W)	
	Same Unit Area[1)]	Different Unit Area[2)]
470	0.38 (380 mA/W)	238
550	0.44 (440 mA/W)	237
665	0.54 (540 mA/W)	262
800	0.65 (650 mA/W)	110
900	0.73 (730 mA/W)	134
1000	0.81 (810 mA/W)	95.8

[1)] Unit areas are calibrated to have the same area for incident light and charge transport.
[2)] Unit areas are NOT calibrated leading to extremely high R_C.

$$R_C = \eta \frac{q}{hf} = \eta \frac{\lambda\ (\mu m)}{1.23985(\mu m \times W/A)}$$

η : Quantum efficiency of the detector for a given wavelength
q : Elementary charge
f : Frequency of the optical signal
h : Planck's constant
λ : Wavelength of the optical signal

Figure S10. Discrepancy of theoretical responsivity in OPTR according to the direction of incident light and electron transport: (a) Illustration for the direction of incident light (unit area = Area_2 = L x W) and charge transport (unit area = Area_1 = T x W) in the channel region. (b) Theoretical responsivity as a function of wavelength in the case of 'Same Unit Area (calibrated)' and 'Different Unit Area (uncalibrated)' (note that the same unit area condition is simply made without calibration in the case of typical photodiode structures).

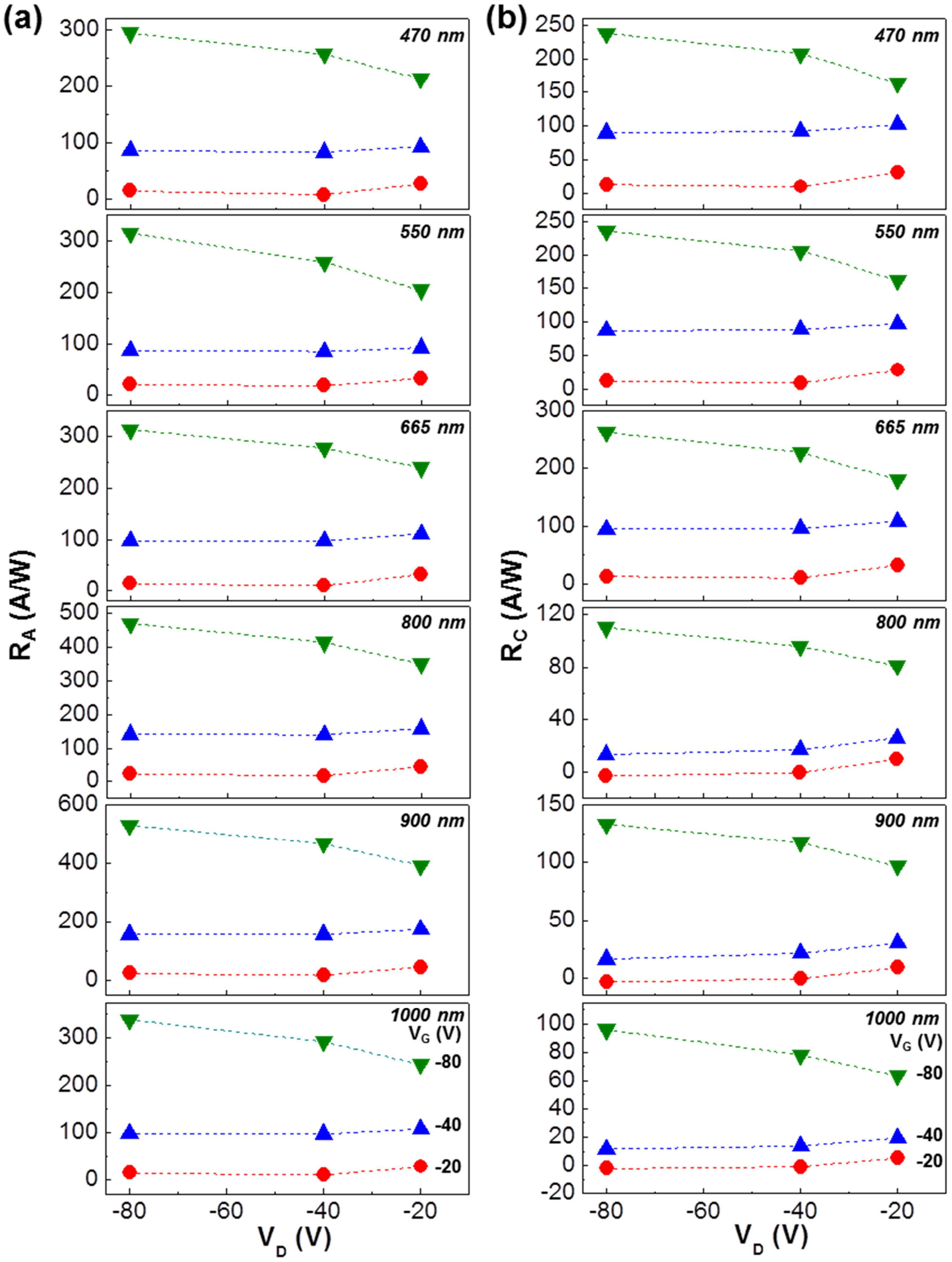

Figure S11. Replotted responsivity (a: R_A and b: R_C) from Fig. S9 in the case of uncalibrated unit areas for incident light and charge transport. Note that the responsivity values without unit area calibration are extremely huge (>2 x 10^5 mA/W at 470 nm) as expected in the table of Fig. S10b.

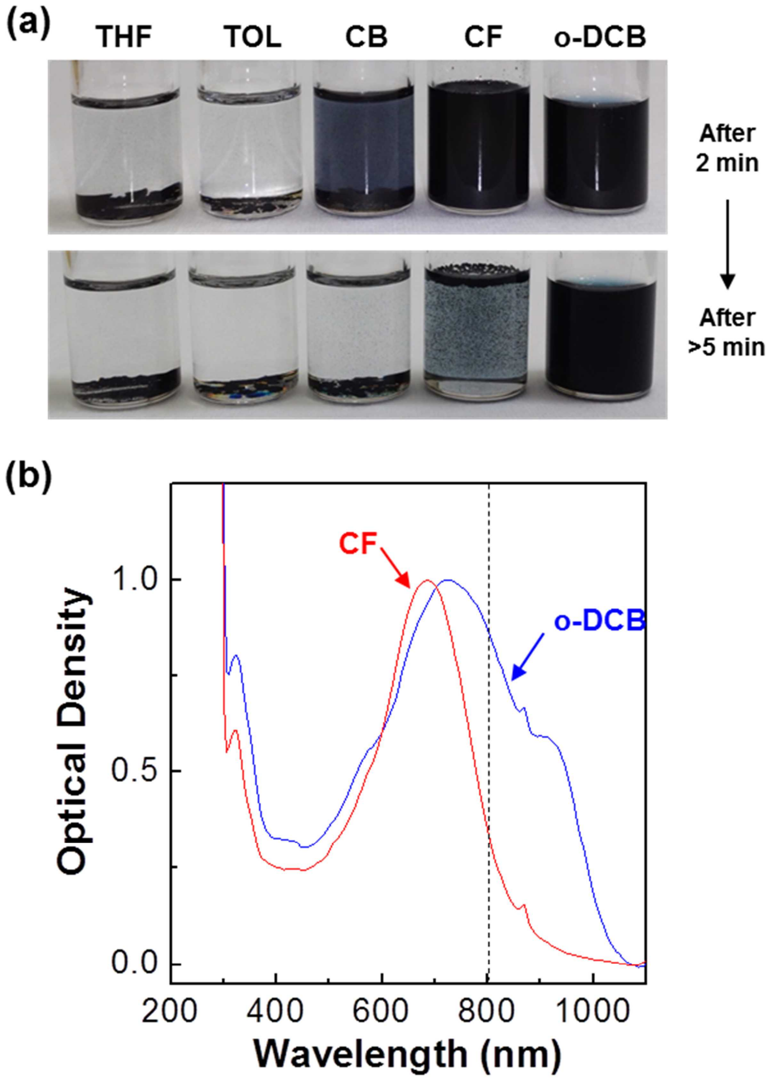

Figure S12. (a) Solubility of PEHTPPD-BT polymer in tetrahydrofuran (THF), toluene (TOL), chlorobenzene (CB), chloroform (CF), and 1,2-dichlorobenzene (o-DCB): (top) images taken in 2 min after stop stirring, (bottom) images taken in >5 min after stop stirring. (b) Comparison of absorption spectra for the PEHTPPD-BT polymer solutions prepared using either CF or o-DCB.

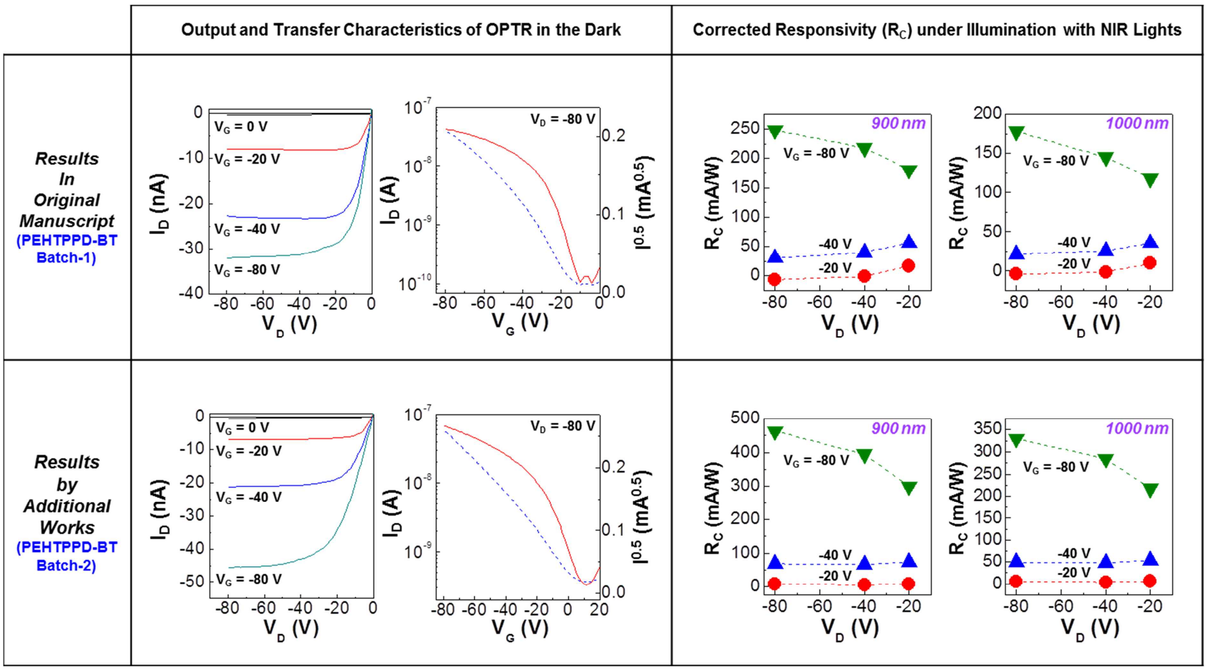

Figure S13. Reproducibility of device results for NIR detection: Two different synthesis batches (PEHTPPD-BT Batch-1 and Batch-2) were used for the fabrication of devices. Note that slightly improved device performances were achieved when Batch-2 was used because of further optimized conditions (skills) for device fabrication.
